# Assessment of Hydrazone Derivatives for Enhanced Steel Corrosion Resistance in 15 wt.% HCl Environments: A Dual Experimental and Theoretical Perspective

**DOI:** 10.3390/molecules29050985

**Published:** 2024-02-23

**Authors:** Abdelilah El-khlifi, Fatima Zahrae Zouhair, Mustafa R. Al-Hadeethi, Hassane Lgaz, Han-seung Lee, Rachid Salghi, Belkheir Hammouti, Hamid Erramli

**Affiliations:** 1 Team of Materials, Electrochemistry and Environment, Laboratory of Organic Chemistry, Catalysis, and Environment, Faculty of Sciences, Ibn Tofail University, BP 133, Kenitra 14000, Morocco; abdoulkhlifi@gmail.com (A.E.-k.); hamid.erramli@uit.ac.ma (H.E.); 2 Laboratory of Plant, Animal and Agro Industry Productions, Faculty of Sciences, Ibn Tofail University, B.P. 133, Kenitra 14000, Morocco; fatima.z.zouhair@gmail.com; 3 Department of Chemistry, College of Education, University of Kirkuk, Kirkuk 36001, Iraq; mustfaa70@yahoo.com; 4 Innovative Durable Building and Infrastructure Research Center, Center for Creative Convergence Education, Hanyang University ERICA, 55 Hanyangdaehak-ro, Sangrok-gu, Ansan-si 15588, Gyeonggi-do, Republic of Korea; 5 Department of Architectural Engineering, Hanyang University ERICA, 55 Hanyangdaehak-ro, San-grok-gu, Ansan-si 15588, Gyeonggi-do, Republic of Korea; ercleehs@hanyang.ac.kr; 6 Euromed Research Center, Euromed Polytechnic School, Euromed University of Fes, Eco-Campus, Fes-Meknes Road, Fes 30030, Morocco; r.salghi@uiz.ac.ma (R.S.); hammoutib@gmail.com (B.H.); 7 Laboratory of Applied Chemistry and Environment, ENSA, University Ibn Zohr, P.O. Box 1136, Agadir 80000, Morocco

**Keywords:** corrosion inhibition, pipeline, computational method, density functional tight-binding, steel, corrosion testing, hydrazone

## Abstract

This study evaluates the corrosion inhibition capabilities of two novel hydrazone derivatives, (E)-2-(5-methoxy-2-methyl-1H-indol-3-yl)-N′-(4-methylbenzylidene)acetohydrazide (MeHDZ) and (E)-N′-benzylidene-2-(5-methoxy-2-methyl-1H-indol-3-yl)acetohydrazide (HHDZ), on carbon steel in a 15 wt.% HCl solution. A comprehensive suite of analytical techniques, including gravimetric analysis, potentiodynamic polarization (PDP), electrochemical impedance spectroscopy (EIS), and scanning electron microscopy (SEM), demonstrates their significant inhibition efficiency. At an optimal concentration of 5 × 10^−3^ mol/L, MeHDZ and HHDZ achieve remarkable inhibition efficiencies of 98% and 94%, respectively. EIS measurements reveal a dramatic reduction in effective double-layer capacitance (from 236.2 to 52.8 and 75.3 µF/cm^2^), strongly suggesting inhibitor adsorption on the steel surface. This effect is further corroborated by an increase in polarization resistance and a significant decrease in corrosion current density at optimal concentrations. Moreover, these inhibitors demonstrate sustained corrosion mitigation over extended exposure durations and maintain effectiveness even under elevated temperatures, highlighting their potential for diverse operational conditions. The adsorption process of these inhibitors aligns well with the Langmuir adsorption isotherm, implying physicochemical interactions at the carbon steel surface. Density functional tight-binding (DFTB) calculations and molecular dynamics simulations provide insights into the inhibitor-surface interaction mechanism, further elucidating the potential of these hydrazone derivatives as highly effective corrosion inhibitors in acidic environments.

## 1. Introduction

Organic compounds, particularly those featuring a large number of heteroatoms and conjugated systems, significantly mitigate the corrosion of carbon steel. Among these, hydrazones stand out for their effectiveness in corrosion inhibition, attributed to their imine linkage R_1_R_2_C=NHNH_2_ and the presence of two nitrogen atoms serving as active adsorption centers [1,2,3,4]. Due to their reactivity towards electrophiles (carbon atoms) and nucleophiles (nitrogen atoms), hydrazones find diverse applications, including as constituents in anti-inflammatory, antimicrobial, muscle relaxant, antihistaminic, antiviral, antituberculous, analgesic, and antibacterial drugs [5,6,7,8]. Moreover, hydrazone derivatives are also employed as corrosion inhibitors in acidic environments [9,10].

On another note, 5-methoxy-2-methyl-3-indolacetic acid (MMIAA), a well-characterized impurity found in the non-steroidal anti-inflammatory drug (NSAID) Indomethacin (IND), has been identified and isolated through various analytical techniques [11,12]. The successful application of synthesized hydrazone could pave the way for the valorization of MMIAA in the development of useful products for corrosion protection. Converting the free carboxylic group in NSAIDs to hydrazone derivatives could mitigate the side effect of gastrointestinal toxicity [8,13].

The efficient transport of resources underpins industrial societies. Industries ranging from energy production to agriculture rely on pipelines to move commodities like petroleum, natural gas, processed foods, and potable water [14]. Unfortunately, corrosion poses a significant threat to the longevity of these metal pipelines, particularly in oil and gas operations where exposure to harsh environments is common [15,16]. Protecting these assets is vital for sustainable operations, making the search for effective corrosion control measures a focus of significant research [17,18,19].

A case in point is the use of strong acids (e.g., 15–20 wt.% HCl) for processes like pipeline descaling and stimulation of oil and gas wells [20,21,22]. While essential for maintaining flow, these acids exacerbate corrosion threats. This highlights the need for reliable corrosion mitigation within such aggressive operating environments.

Carbon steel, prized for its mechanical properties, finds extensive use in industrial infrastructure [23,24]. However, its inherent vulnerability to acids necessitates protective measures [15]. Researchers have explored various corrosion prevention strategies, including cathodic protection, the application of protective coatings, and the development of corrosion inhibitors [17,18,19].

The effectiveness of corrosion inhibitors in reducing or preventing corrosion on carbon steel surfaces has been well established for many years. Their remarkable corrosion resistance, cost-effectiveness, and ease of application in industries make them particularly attractive [25,26,27]. These inhibitors are categorized into different classes based on their mechanism of action and chemical structure: anodic, cathodic, or mixed for the former criterion, and inorganic or organic for the latter [28]. Functional groups present in organic compounds, such as methoxy, hydroxyl, aromatic rings, heteroatoms, etc., create adsorption sites and enhance the bond between the metal and the corrosion inhibitor through electron exchange [29]. Generally, organic corrosion inhibitors act by blocking active areas on the metal surface via physical, chemical, or physicochemical interactions with it. However, it is reported that the adsorption of these organic compounds is not merely physisorptive or chemisorptive, but follows a complex adsorption mechanism combining both [30].

Density Functional Tight Binding (DFTB) emerges as a pivotal computational technique, especially in the context of analyzing the interaction between corrosion inhibitors and metal surfaces [31,32]. It is a computationally efficient variant of Density Functional Theory (DFT) that stands out for its ability to model electronic structures with remarkable accuracy, yet at a fraction of the computational cost associated with traditional DFT methods [33]. This makes it exceptionally well-suited for probing the complex adsorption phenomena of organic inhibitors onto metal substrates [34]. By offering insights into the molecular interactions and electron density distributions at the inhibitor-metal interface, DFTB aids in deciphering the intricate mechanisms governing corrosion inhibition, thus driving the innovative design of more effective and tailored corrosion-resistant materials.

This study investigates the potential of two novel hydrazone derivatives, (E)-2-(5-methoxy-2-methyl-1H-indol-3-yl)-N′-(4-methylbenzylidene)acetohydrazide (MeHDZ) and (E)-N′-benzylidene-2-(5-methoxy-2-methyl-1H-indol-3-yl)acetohydrazide (HHDZ), for corrosion inhibition of N80 carbon steel in a harsh 15 wt.% HCl environment. A multi-faceted experimental approach was employed, including weight loss measurements, potentiodynamic polarization (PDP), and electrochemical impedance spectroscopy (EIS). These techniques enabled the assessment of inhibitor performance across varying concentrations and exposure times. To visualize surface effects, scanning electron microscopy (SEM) provided a detailed examination of the steel morphology before and after inhibitor treatment. For a deeper mechanistic understanding, computational techniques were integrated into the analysis. Density Functional Tight Binding (DFTB) simulations shed light on the bonding interactions between the hydrazone molecules’ active sites and the iron atoms on the steel surface. This computational insight contributed to a comprehensive picture of the inhibition mechanism. Further, molecular dynamics simulations revealed the likely adsorption configurations of the inhibitor molecules on the Fe(110) surface in an aqueous environment.

## 2. Results and Discussion

### 2.1. Weight Loss: Effect of Concentration and Temperature

The weight loss method remains one of the most precise and direct approaches for measuring metal corrosion rates and the effectiveness of a corrosion inhibitor. It constitutes a direct corrosion monitoring method that provides data on average corrosion rates [35]. This method was employed to investigate the corrosion of carbon steel at 303 K and 333 K in a 15 wt.% HCl solution, with and without the tested hydrazones MeHDZ and HHDZ. Table 1 presents the results of the weight loss measurements.

The findings demonstrate a significant reduction in the weight loss and corrosion rate of carbon steel in the presence of the inhibitors. Even at a low concentration (10^−4^ mol/L) in the acidic solution, the inhibitors effectively reduced the corrosion rate of the steel sample, decreasing from 23.7 mm/year to 2.315 and 2.744 mm/year for MeHDZ and HHDZ, respectively, at 303 K. This indicates that the tested hydrazones in the 15 wt.% HCl solution act as highly effective corrosion inhibitors for carbon steel. With increasing inhibitor concentration, the corrosion rate continued to decrease, reaching a minimum of 0.85 and 1.509 mm/year at 5 × 10^−3^ mol/L. The potent inhibitory properties of these compounds can be attributed to the strong interaction between their molecules and the steel surface. These compounds contain various potential interaction centers such as O, N, and aromatic rings. These centers can form strong bonds with the steel surface, thereby preventing metal corrosion. The corrosion rate of the steel was significantly reduced in the presence of the inhibitors, and the inhibitory effect was superior at higher concentrations, reaching a maximum of 96.41% and 93.63% at 5 × 10^−3^ mol/L for MeHDZ and HHDZ, respectively, at 303 K.

The influence of temperature on the anticorrosive effect of various inhibitor concentrations was investigated by conducting additional weight loss experiments at elevated temperatures (333 K). As anticipated, the corrosion rate of the carbon steel sample increased with temperature, both in inhibited and uninhibited solutions. However, compared to the rate observed in the uninhibited acidic solution, the corrosion rate on the carbon steel sample immersed in hydrazone-inhibited solutions was notably lower (Table 1). This result suggests that the hydrazone molecules studied are effective in suppressing steel corrosion at the tested temperatures [36,37,38]. Nonetheless, the inhibitory effect diminished with an increase in temperature. This behavior is primarily associated with the contribution of physical interactions. Physical forces are weaker than chemical bonds and can be disrupted by higher temperatures, leading to partial desorption of the inhibitor molecules, thereby reducing inhibition efficiency.

The high inhibition efficiency observed at elevated temperatures (90.56% and 87.68% at 5 × 10^−3^ mol/L for MeHDZ and HHDZ, respectively, at 333 K) suggests that chemical adsorption plays a dominant role in the inhibition process, given that inhibitor performance remains high even at increased temperatures. This indicates that the interactions between the inhibitors and the carbon steel surface are not solely governed by physical forces but also involve significant chemical bonding [39,40,41]. Chemical adsorption, characterized by the formation of covalent or ionic bonds between the inhibitor molecules and the metal surface, is generally more resistant to temperature fluctuations. This enhanced resistance enables the inhibitors to maintain their effectiveness even under more thermally demanding conditions, thereby highlighting their potential for applications in environments where temperature is a critical factor.

### 2.2. Adsorption Isotherm Assessment

**Figure 1:**
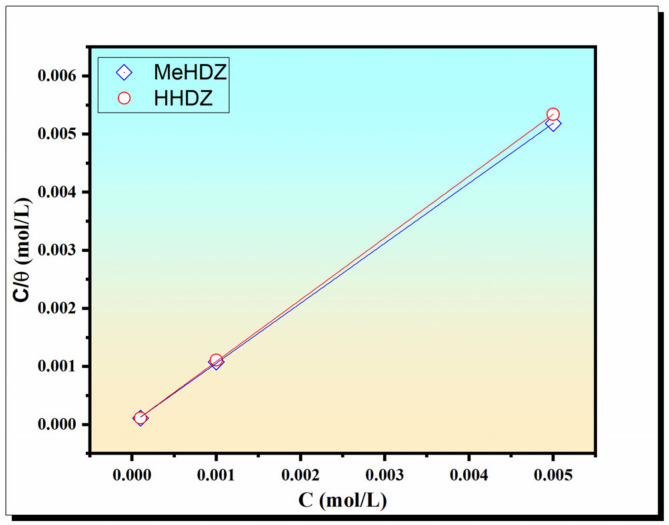
Langmuir adsorption isotherm of MeHDZ and HHDZ on the carbon steel surface at 303 K.

Inhibitor molecules protect metal surfaces from corrosion by forming protective barriers. These barriers, which may consist of one or more molecular layers, are established when inhibitor molecules attach to the metal surface through physical or chemical means, or a combination of both [20]. Among the various isotherm models studied to understand the adsorption of hydrazones on the steel surface, the Langmuir adsorption isotherm model proved to be the most accurate and appropriate. According to this model, the adsorbate molecules attach to the metal surface in the form of a single, homogenous, and independent layer [42]. The graphical representation of inhibitor concentration (C) versus the ratio of C to the extent of surface coverage (θ), (C/θ), should yield a linear relationship. The slope of this curve measures the maximum amount of the inhibitors’ molecules that can be adsorbed on the metal surface. The intercept of the curve equals 1/K_ads_. Linear plots were obtained from gravimetric measurement parameters (Figure 1). Table 2 presents the values of parameters obtained from the analysis of the plot.

A physisorptive adsorption mechanism is proposed when the values of ${∆G}_{ads}^{{}^{\circ}}$ are equal to or less than −20 kJ/mol, while a chemisorptive adsorption mechanism is suggested for values ranging from −20 to −40 kJ/mol [43,44]. In the range of −20 to −40 kJ/mol, a mixed mechanism involving both physisorption and chemisorption is suggested [45]. By analyzing the thermodynamic data, the ${∆G}_{ads}^{{}^{\circ}}$ values of the inhibitors were determined to be −36.79 and −36.83 kJ/mol for MeHDZ and HHDZ, respectively. These values, situated in the range of −20 to −40 kJ/mol, indicate that the hydrazones are adsorbed onto the steel surface via a mixed adsorption mechanism, combining both chemisorption and physisorption. The negative values of ${∆G}_{ads}^{{}^{\circ}}$ demonstrate the stability and spontaneity of the adsorption process [46].

### 2.3. Electrochemical Study

#### 2.3.1. Potentiodynamic Polarization Curves

**Figure 2:**
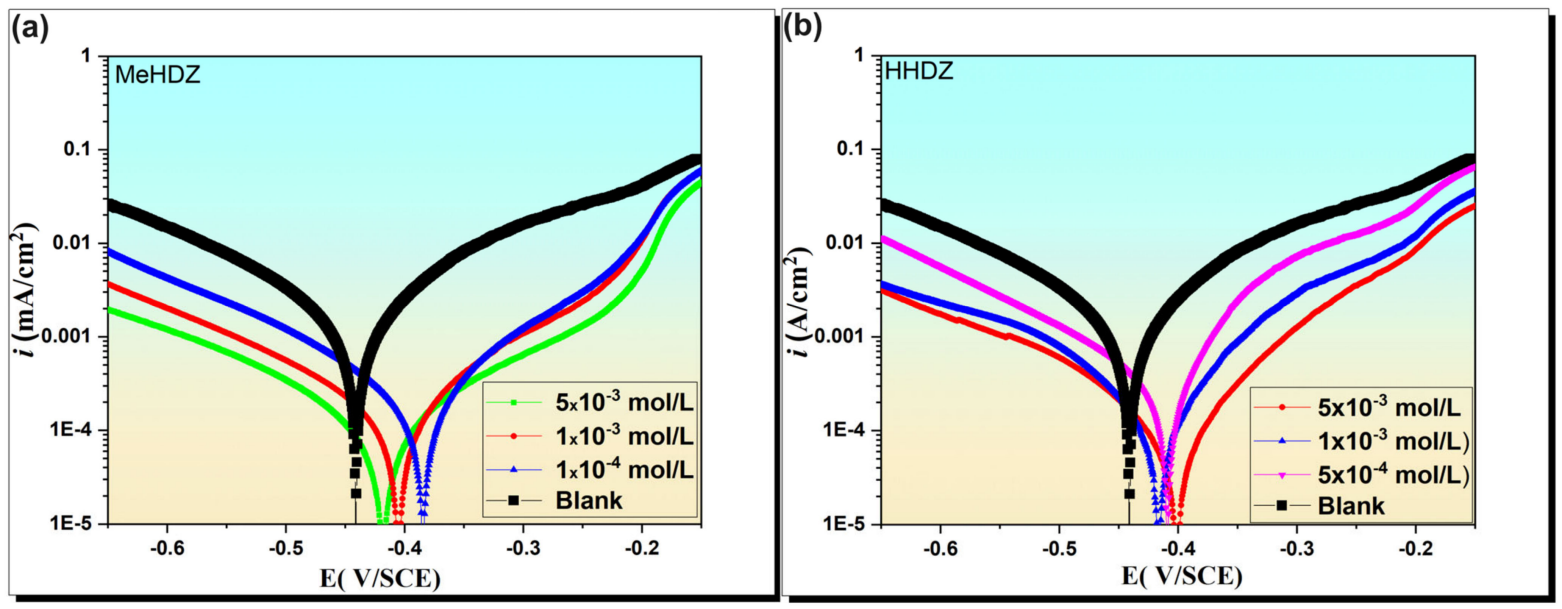
Polarization curves of carbon steel in 15 wt.% HCl without and with the addition of different concentrations of (**a**) MeHDZ and (**b**) HHDZ at 303 K.

The potentiodynamic polarization curves for carbon steel in a 15 wt.% HCl solution, with and without various concentrations of MeHDZ and HHDZ inhibitors, are depicted in Figure 2. It is observed that for both corrosion inhibitors, the polarization curves exhibit almost identical shapes, suggesting a similar inhibition mechanism where the inhibitors likely act by reducing the rates of both hydrogen reduction and steel dissolution, without fundamentally altering the underlying corrosion processes. Compared to the control test, increasing the concentration of inhibitors resulted in a shift of the cathodic and anodic potentiodynamic polarization curves towards a lower current density, indicating that both MeHDZ and HHDZ inhibitors have a significant inhibitory effect on the cathodic and anodic reactions of the steel electrode in acidic solution. Comparing the slope of the cathodic polarization curve, the nearly parallel cathodic polarization curve indicates that the two inhibitors do not impact the mechanism of the cathodic reaction, and that the cathodic reaction is still controlled by the hydrogen evolution reaction [47].

Based on the potential shift from E_corr_, inhibitors can be categorized into three types: anodic, cathodic, or mixed. A mixed-type inhibitor is one that shows a minor shift in either direction. Cathodic inhibitors are indicated by a significant shift of potential towards the cathodic direction, while anodic inhibitors are indicated by a significant shift towards the anodic direction [48,49]. The results from the PPC study suggest that the inhibitor acts as a mixed-type inhibitor. Both the cathodic and anodic branches of the current density value are shifted to a lower value compared to that of the control.

The PPC parameters derived from the curve fitting, including corrosion current density (*i*_corr_), corrosion potential (*E*_corr_), Tafel anodic (*β*_a_) and cathodic (*β*_c_) slopes, as well as the calculated inhibition efficiency *η* (%) are presented in Table 3. As per Table 3, it can be observed that, compared to the control test, the addition of inhibitors leads to a very low corrosion current density, and as the concentration of inhibitors increases, the corrosion current density decreases significantly, and corrosion efficiency increases. The corrosion current density and the maximum inhibition efficiency are 95 and 118 (µA/cm^^2^^) (control is 1711 µA/cm^^2^^) and 94% and 93% for MeHDZ and HHDZ, respectively. This demonstrates that both inhibitors have an excellent inhibitory effect, and the results are consistent with the experimental findings from the weight loss measurements.

#### 2.3.2. EIS Studies: Effect of Concentration

**Figure 3:**
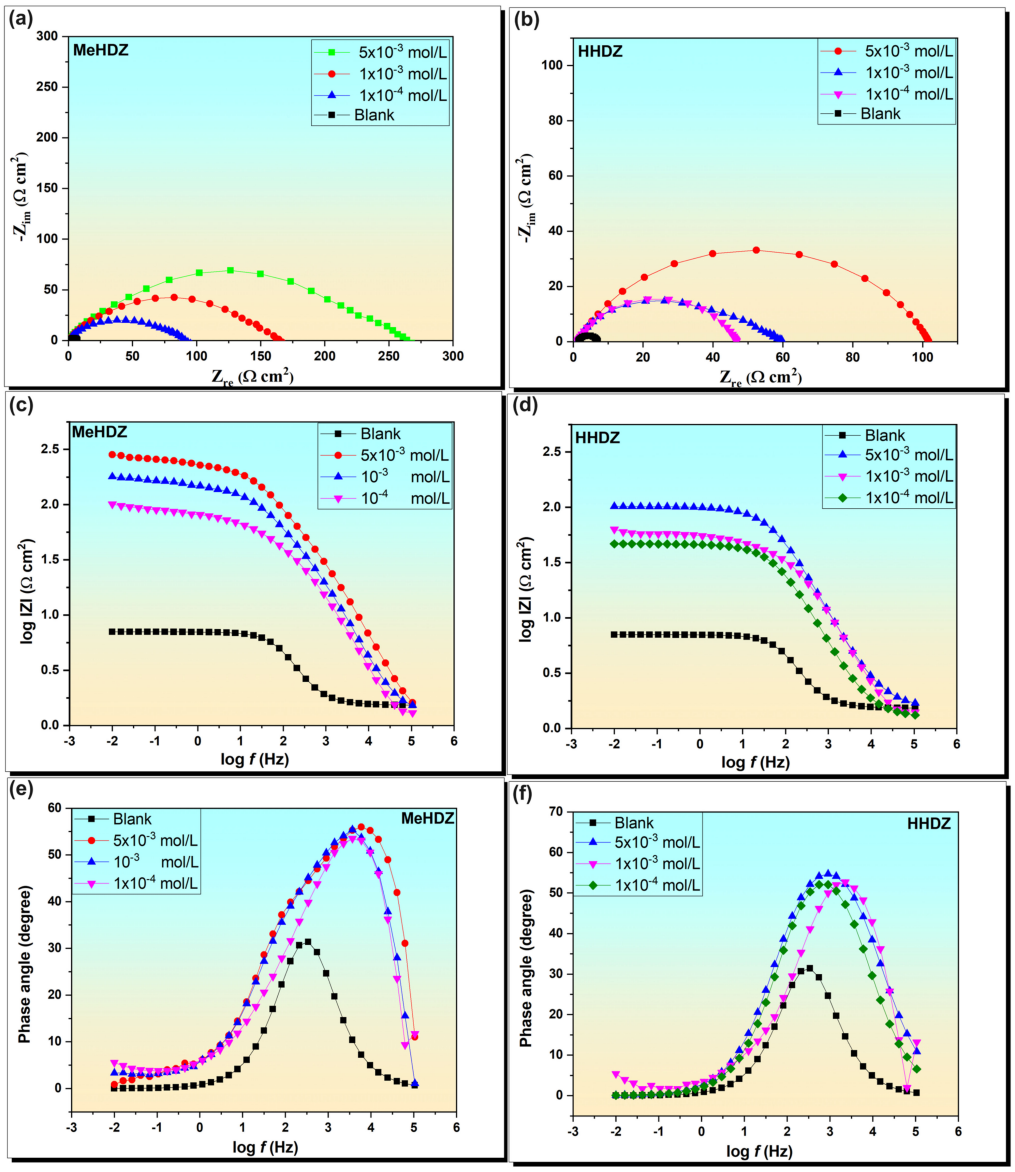
Impedance diagrams of carbon steel in 15 wt.% HCl without and with the addition of different concentrations of MeHDZ and HHDZ at 303 K. (**a**,**b**) Nyquist plots and (**c**–**f**) Bode diagrams.

The corrosion behavior of carbon steel in a 15 wt.% HCl solution in the absence and presence of hydrazone inhibitors was investigated using the method of EIS at 303 K. The corresponding Nyquist plots and Bode diagrams, without and with various concentrations of MeHDZ and HHDZ, are presented in Figure 3.

The Nyquist plots exhibit single semi-circles with their center positioned below the real axis, indicating that the corrosion processes of carbon steel are also associated with frequency dispersion, surface roughness, and inhomogeneity of the surface electrode [50,51]. The addition of inhibitors does not alter the shape of the impedance, which suggests that the presence of MeHDZ and HHDZ in the corrosive solution does not change the corrosion mechanism of carbon steel in 15 wt.% HCl. However, the diameter of this semi-circle increases in the presence of inhibitors, implying the formation of more integrated protective adsorption layers with the increase in inhibitor concentration [52,53]. This also indicates that as the concentration of inhibitors increases, a greater number of molecules adsorb onto the steel surface within the electrical double layer, forming a protective barrier that hinders the transport of corrosive species to the metal surface.

The addition of hydrazones to the acidic corrosive environments increases impedance and phase angle (Figure 3c–f), indicating corrosion inhibition. Inhibited solutions demonstrate larger Bode phase values and a more pronounced linear portion in the Bode modulus diagrams than the control solutions (blank). This reflects the trends observed in the Nyquist plots. There is a marked shift towards higher values of |Z| in the inhibited solutions, suggesting increased surface coverage and protection, likely due to increased adsorption of molecules [54,55].

**Figure 4:**
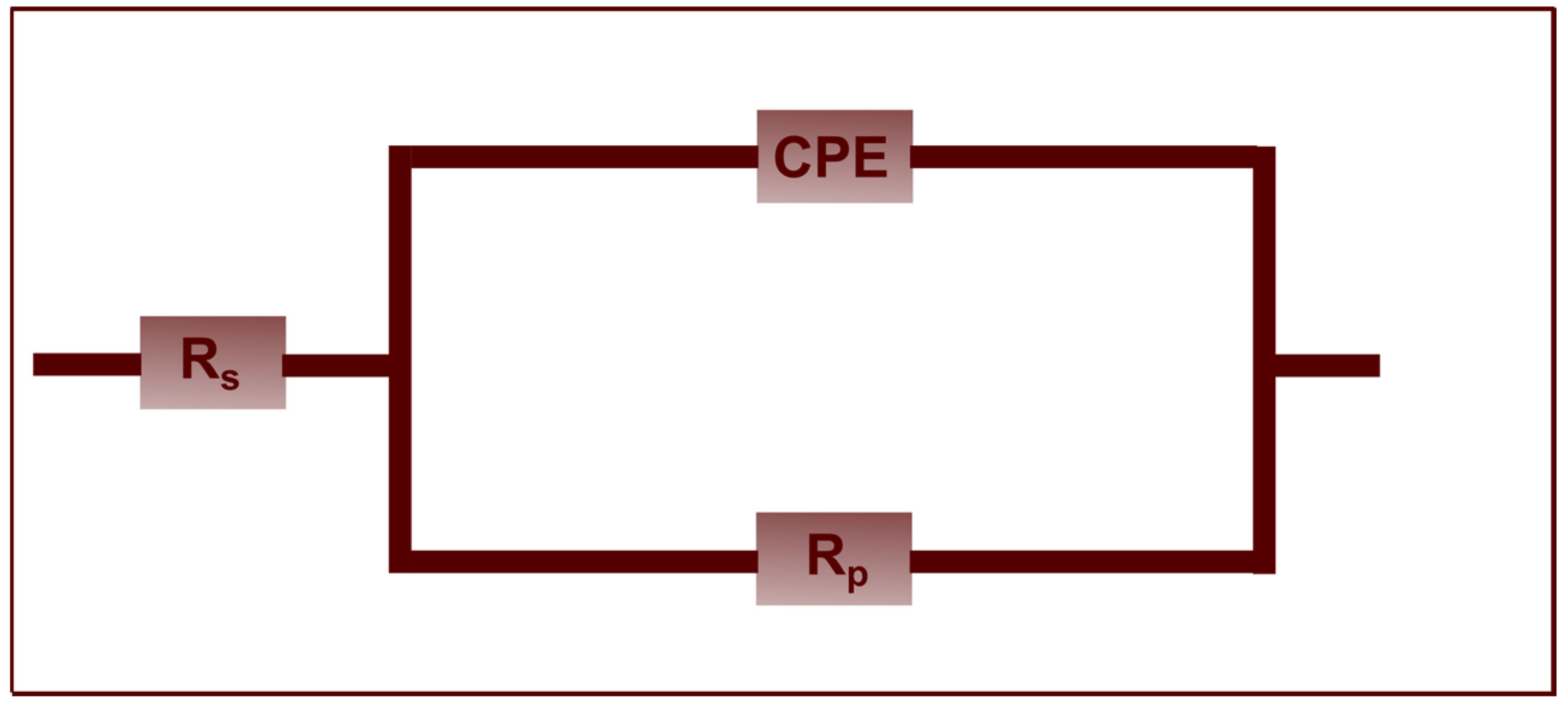
The equivalent circuit model used to fit the steel/15 wt.% HCl interface and the steel/15 wt.% HCl + hydrazones interface.

The impedance spectra were analyzed by fitting the Nyquist plots to an equivalent circuit model, which is shown in Figure 4. The fitted data are listed in Table 4. *R*_s_ is the solution resistance, *R*_p_ is the polarization resistance, which is the sum of all resistances involved, and *Q*_dl_ is the constant phase element (CPE) of the double layer. CPE is a constant phase element to replace the capacitor in the electrochemical process to handle the non-ideal capacitive response [56,57,58,59]. The effective double layer capacitance values can be calculated using Brug’s equation [60,61,62]:

(1) $$C_{eff,dl}=Q^{\frac{1}{n}}\times {\left(\frac{1}{R_{s}}+\frac{1}{R_{P}}\right)}^{\frac{n-1}{n}}$$

where parameters *Q* and *n* are the CPE components.

In Table 4, the values of *Q*_dl_ and *C*_eff,dl_ decrease in the presence of MeHDZ and HHDZ, likely due to a decrease in local dielectric constant and/or an increase in the thickness of the protective layer at the electrode surface [63]. In other words, the decrease in *Q*_dl_ and *C*_eff,dl_ values is attributed to the progressive replacement of water molecules adsorbed on the metal surface by inhibitor molecules with a lower dielectric constant [64]. According to the Helmholtz model of the double layer capacitance, *C*_eff,dl_ is directly proportional to the local dielectric constant ε:

(2) $$C_{dl}=\frac{\varepsilon_{0}\varepsilon S}{d}$$

where d is the thickness of the double layer, S is the total electrode surface area, ε_0_ is the permittivity of air, and ε is the local dielectric constant.

The replacement of water molecules by adsorbed inhibitor molecules would result in a decrease in the value of *ε* and/or an increase in the value of d, leading to a lower value for *C*_eff,dl_ [65].

It is evident from Table 4 that the phase shift value (n) is around 0.80–0.83 and does not change significantly after the addition of various concentrations of inhibitors. This means that the charge transfer process controls the dissolution process both in the absence and presence of various concentrations of inhibitors. The corrosion rate is inversely proportional to *R*_p_. In the presence of both inhibitors, the values of *R*_p_ increase, which is attributable to the formation of a protective film at the metal-solution interface [66]. Moreover, inhibition efficiencies increase with increasing concentrations of inhibitors, reaching 98% and 94% at 5 × 10^−3^ mol/L for MeHDZ and HHDZ, respectively.

The slight difference in corrosion inhibition performance between MeHDZ and HHDZ is mainly attributed to the effect of the additional methyl group in MeHDZ compared to HHDZ. Indeed, the presence of this methyl group in the molecular structure of MeHDZ could influence how the inhibitor interacts with the metal surface, thus altering the efficiency of inhibition. This assertion could be further confirmed by theoretical simulations.

#### 2.3.3. EIS Studies: Effect of Immersion Time

**Figure 5:**
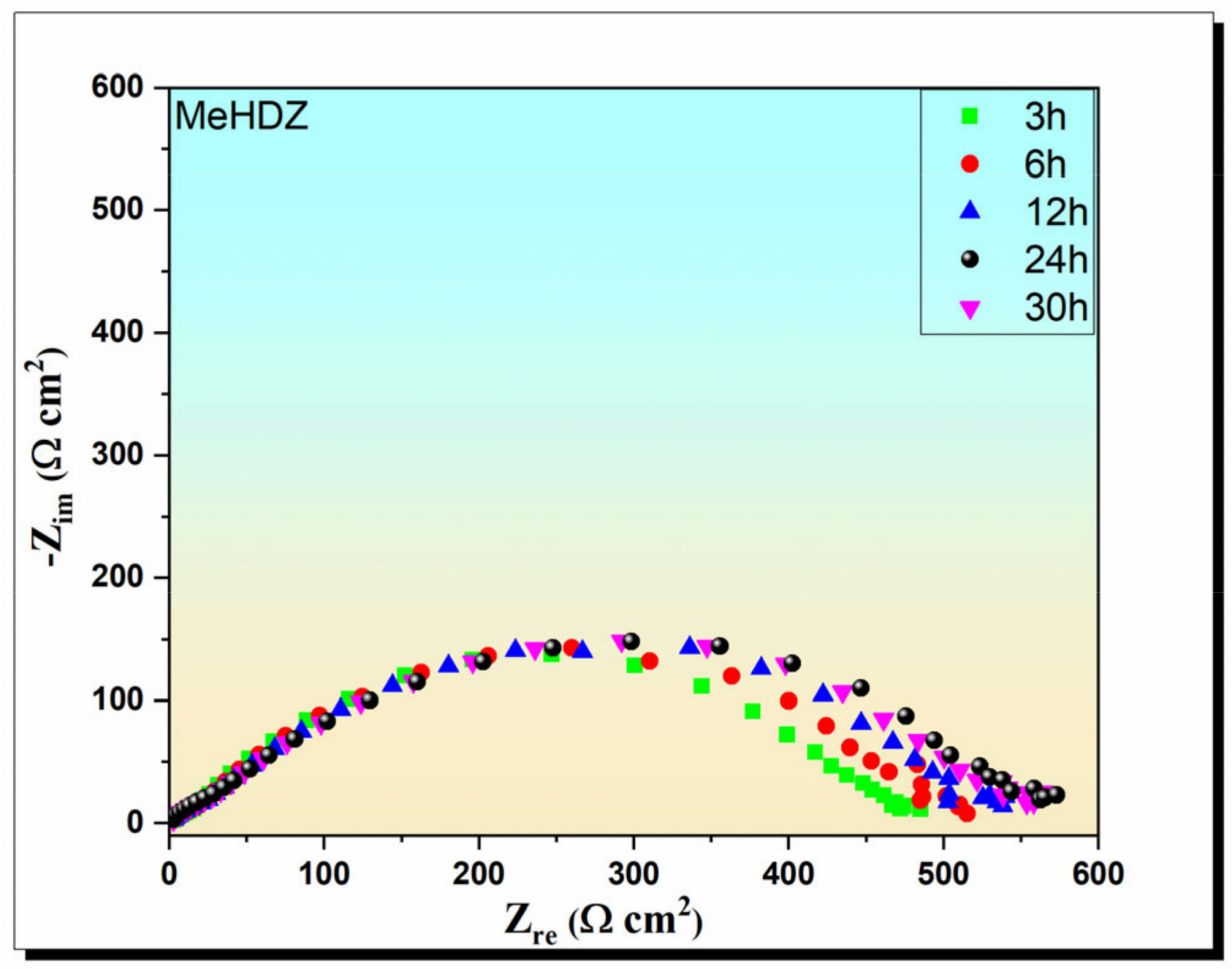
Results of EIS for carbon steel in 15 wt.% HCl with 5 × 10^−3^ mol/L of MeHDZ at different immersion times.

The use of the EIS technique facilitated the assessment of MeHDZ’s effectiveness on carbon steel in a 15 wt.% HCl environment by examining various immersion time intervals ranging from 30 min to 30 h. The concentration of 5 × 10^−3^ mol/L of MeHDZ was selected due to its superior efficacy, as determined by prior experiments. The results of this analysis, depicted by the Nyquist plots, are presented in Figure 5, while the associated electrochemical details are compiled in Table 5.

In the initial hours of immersion, up to 3 h, a moderate increase in impedance is observed, reaching 479 Ω cm^2^. This increase continues and becomes more pronounced after 6 h of immersion. A detailed examination of the information in Table 5 indicates that prolonging the immersion leads to an increase in polarization resistance (*R*_p_) and a reduction in the effective double layer capacitance (*C*_eff’dl_). This decrease in *C*_eff’dl_ suggests a thickening of the electrical double layer, resulting from the attachment of MeHDZ molecules on the steel surface, thereby forming a protective film [55,67,68]. This protective layer gradually densifies, enhancing the steel’s resistance to corrosion. An immersion duration of 24 h appears to be optimal for achieving effective corrosion protection. In conclusion, these results demonstrate that prolonged immersion durations are associated with a significant improvement in corrosion resistance, a crucial element for effectiveness in acidification processes and other industrial applications.

### 2.4. SEM: Surface Characterization

**Figure 6:**
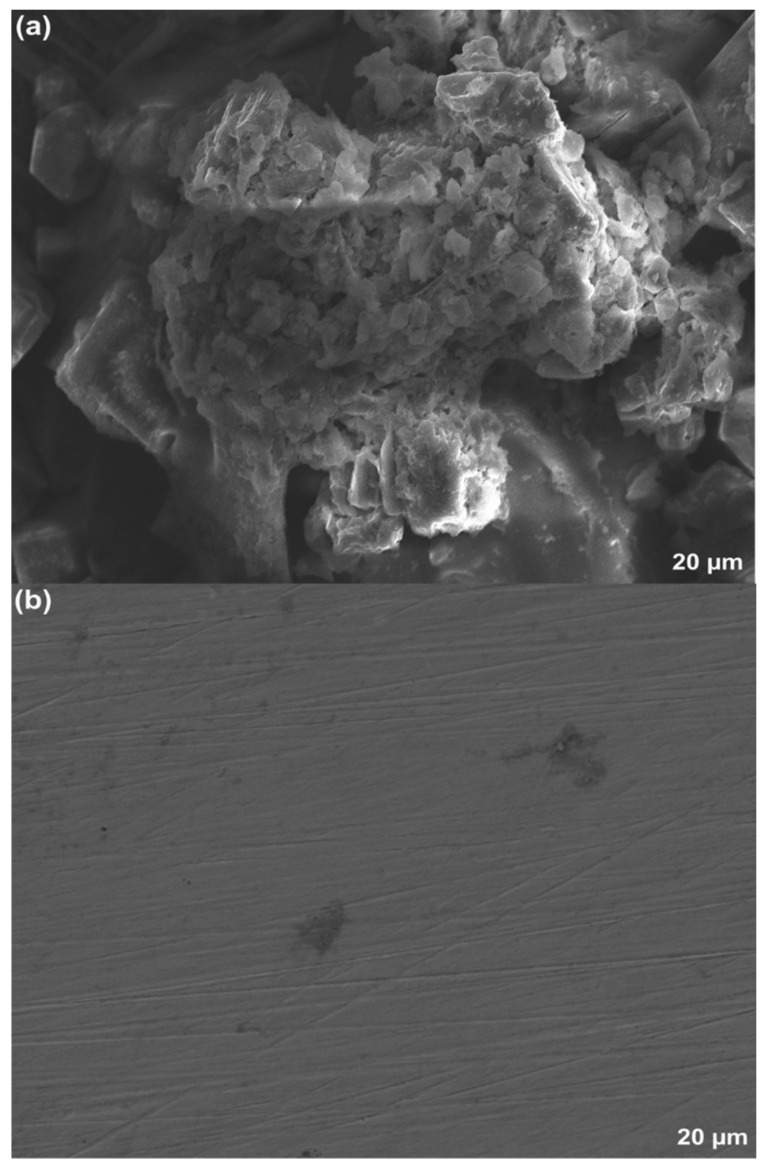
SEM Micrographs of carbon steel in the absence (**a**) and presence of MeHDZ (**b**) at a concentration of 5 × 10^−3^ mol/L at 303 K.

Scanning Electron Microscopy (SEM) provides a detailed approach to study the micromorphology of surfaces, thus enabling precise visualization of changes induced by different treatments. This technique was utilized to examine the microscopic appearance of carbon steel samples after a 24-h immersion in a 15 wt.% HCl solution, with and without the MeHDZ inhibitor, at a temperature of 303 K, as depicted in Figure 6. Figure 6a reveals a severely corroded and heavily damaged metal surface, resulting from the aggressive attack of the HCl solution on the steel. In contrast, the metal surfaces in the presence of the inhibitor, as shown in Figure 6b, appear smooth in comparison with the acidified control group, thereby demonstrating that the inhibitor provide a remarkable inhibitory effect for carbon steel in a highly acidic environment.

SEM analysis highlights the protective effects of the MeHDZ inhibitor, revealing significantly less altered and more uniform metal surfaces in its presence. These observations confirm the efficacy of the inhibitors used in significantly reducing the corrosion of carbon steel under severe acidic conditions.

### 2.5. SCC-DFTB Simulations

**Figure 7:**
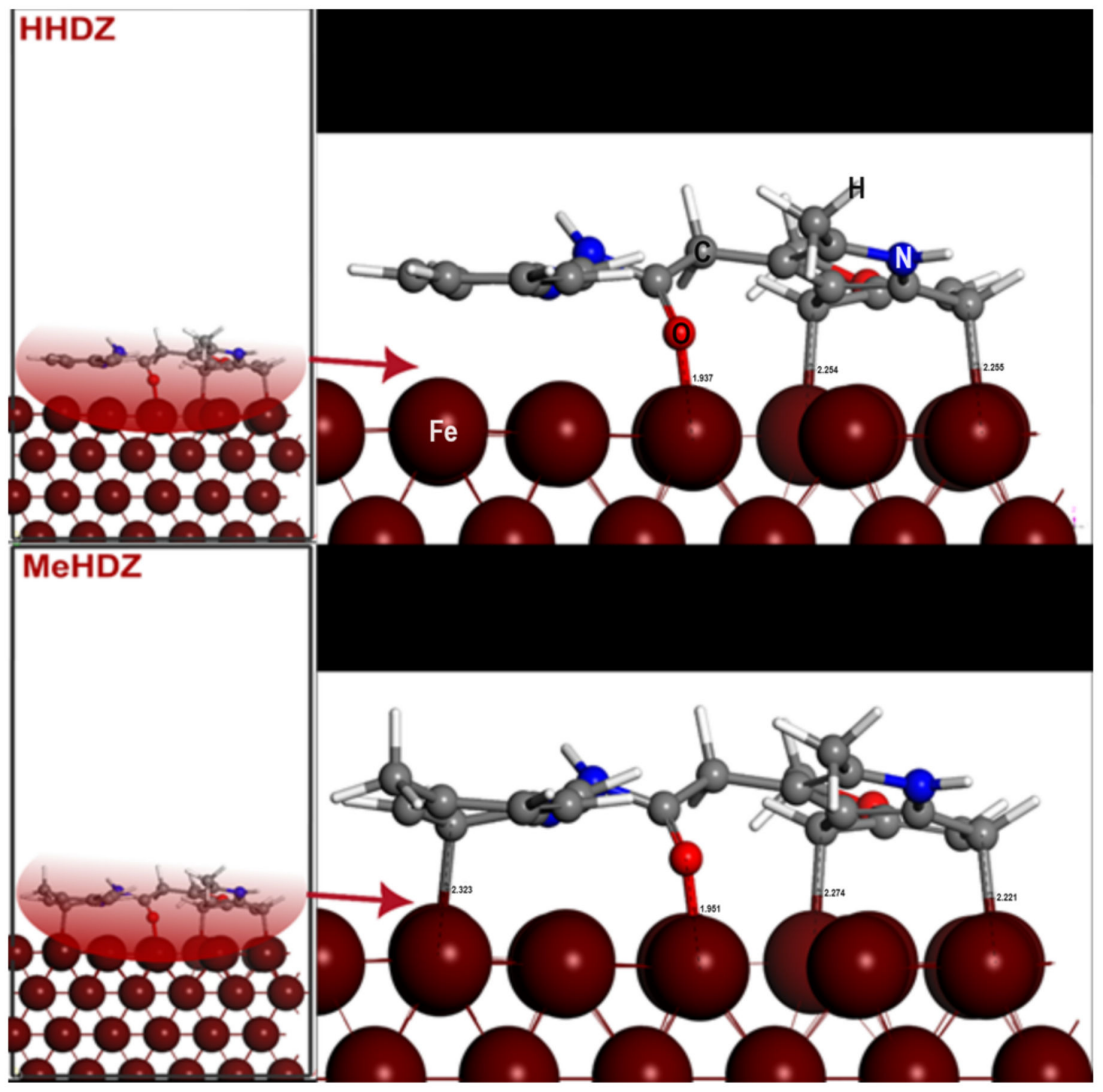
SCC-DFTB optimized adsorption structures of MeHDZ and HHDZ molecules on the Fe(110) Surface. The bond lengths are indicated in angstroms (Å).

The use of SCC-DFTB simulations, an efficient and reliable method for elucidating atomic-scale behavior of molecule-metal adsorption systems, provides reliable, accurate, and scientifically significant insights [69]. In this context, the adsorption process and interactions of MeHDZ and HHDZ molecules on iron surfaces were evaluated using DFTB simulations. Figure 7 illustrates the DFTB-optimized adsorption configurations of MeHDZ and HHDZ on the Fe(110) surface.

It is well established that inhibitory molecules can bind to metal surfaces via complex physicochemical adsorption [45]. Generally, the sum of covalent radii of atoms involved in interactions typically reflects the formation of a chemical bond if the interatomic distance is less than this sum [70]. Conversely, physical interactions tend to manifest at interatomic distances greater than 3 Å [70]. Examining the optimized adsorption configurations for MeHDZ and HHDZ molecules, it is evident that their configuration on the iron surface is similar. Both molecules are in close contact with the top layer of the Fe(110) surface, forming bonds with its atoms. The HHDZ molecule stabilizes in a configuration parallel to the surface through one Fe-O bond and two Fe-C bonds. The Fe-O bond has a length of 1.937 Å, while the two Fe-C bonds are at a distance of 2.254 to 2.255 Å. The MeHDZ molecule, in contrast, stabilizes parallel on the iron surface, forming Fe-O and three Fe-C bonds with lengths of 1.951 Å and 2.221–2.323 Å, respectively.

A deeper understanding of the nature of bonds formed between the hydrazone molecules and iron atoms can be gained by correlating bond distances with the sum of covalent radii of interacting atoms [71]. Previous research has indicated that the cumulative covalent radii for Fe-C (r_C_ + r_Fe_) and Fe-O (r_O_ + r_Fe_) bonds are 2.08 Å and 1.98 Å, respectively [72]. Comparing these with the calculated bond distances, it becomes apparent that the lengths of Fe-C and Fe-O bonds fall within the sum of the corresponding covalent radii, suggesting potential chemical interactions between the atoms of the molecules and the iron surface.

Assessing interaction energies derived from the optimized adsorption configurations can shed more light on the adsorption potency of MeHDZ and HHDZ molecules on the iron surface. As indicated in the figure for each optimized structure, MeHDZ exhibits a large magnitude of interaction energy, suggesting its greater adsorption capabilities, followed by HHDZ having the least magnitude of interaction energy. MeHDZ and HHDZ systems present negative interaction energies of −2.241 and −2.057 eV, respectively, suggesting their thermodynamically favored adsorption and propensity for interactions with iron atoms [73,74]. These results highlight the excellent adsorption characteristics of the MeHDZ compound compared to HHDZ, as it is more inclined to coordinate with iron atoms. Given these results, it can be inferred that the methyl group plays a decisive role in the individual adsorption of molecules. That said, long-range dispersion forces can nonetheless exert a considerable influence on the interactions between molecules and surfaces and might contribute to its interaction energy [75]. Experimental investigations primarily suggest complex interactions involving both physical and chemical interactions, notably contributing to the stability of the inhibitory layer formed on the metal surface in acidic environments [76,77,78].

### 2.6. Molecular Dynamics Simulations

**Figure 8:**
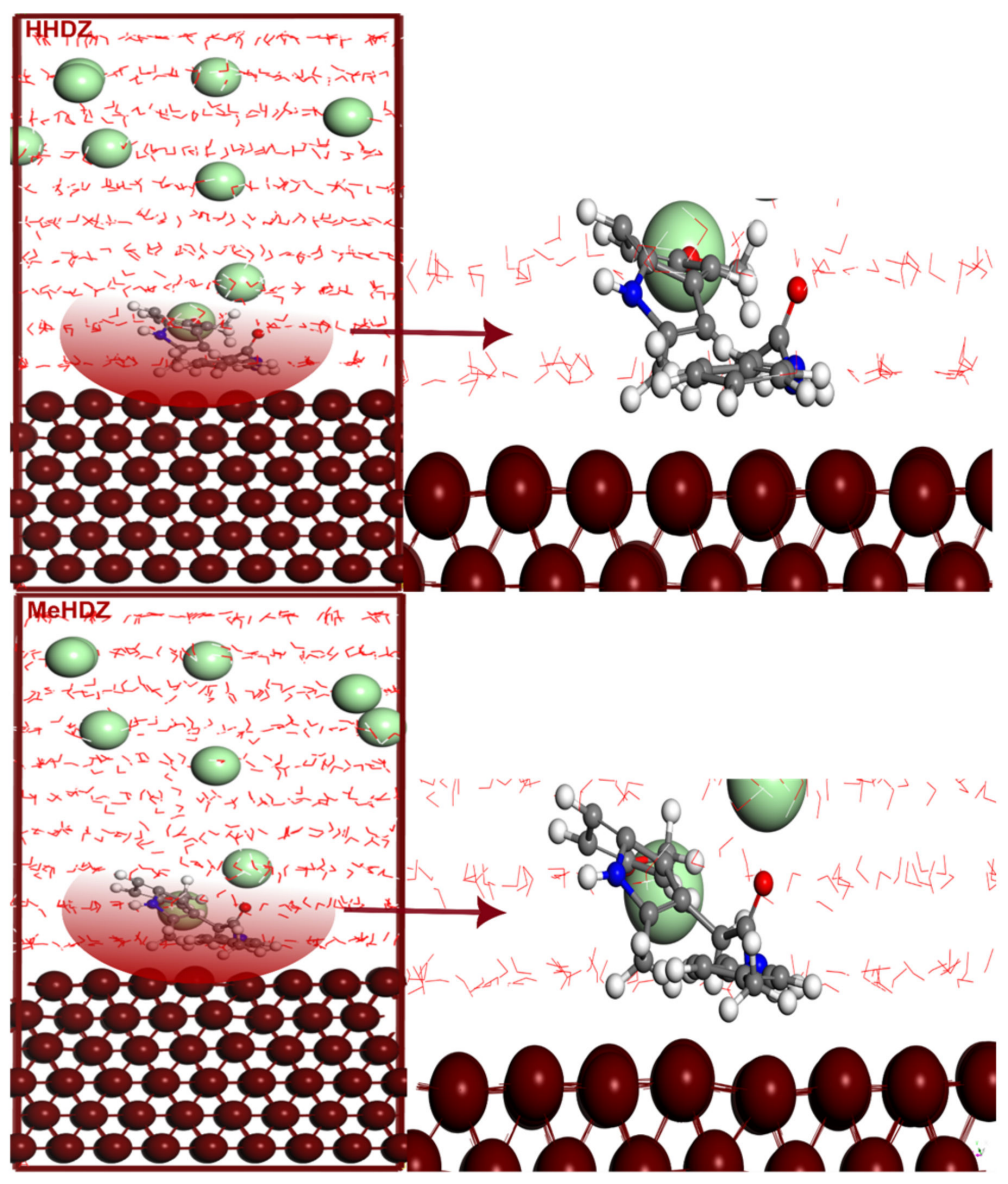
Most thermodynamically stable adsorption configurations for HHDZ and MeHDZ molecules on the Fe(110) surface in aqueous environment, as derived from MD simulations.

Molecular dynamics (MD) is a powerful computational tool for analyzing molecular adsorption on metal surfaces, allowing a detailed understanding of atomic and molecular interactions. This method accounts for all interactions within the system, including those between the molecules and the surface. MD simulations reveal valuable insights into the configuration and adsorption behavior of molecules in aqueous environments [34]. Herein, MD simulations were carried out to identify the most stable adsorption configurations of MeHDZ and HHDZ molecules on the iron surface at 303 K, in the presence of a solvent layer. These configurations are illustrated in Figure 8.

The results of the adsorption configuration indicate that both molecules, MeHDZ and HHDZ, tend to align parallel to the iron surface, primarily through their functional group and the hydrazone moiety. However, the methoxyindole part of these molecules is oriented towards the solvent layer. This orientation of molecules suggests a high likelihood of electron exchange between the molecules and iron, where the phenyl/methylphenyl groups and hydrazone sites could act as electron donors to the vacant orbitals of the metal. Therefore, a comparative assessment of the adsorption energies of each inhibitor is crucial for a better understanding of their efficacy.

As expected, the MeHDZ molecule displays a significantly high adsorption energy of −205.14 kcal/mol, surpassing that of HHDZ, which is −191.21 kcal/mol. This difference could be attributed to the presence of an additional methyl group in MeHDZ, increasing its affinity for the iron surface. Nevertheless, both molecules exhibit negative adsorption energies, indicating their spontaneous and favorable adsorption on the iron surface [79]. These observations from molecular dynamics simulations reinforce the hypotheses and conclusions derived from experimental results, thus highlighting the effectiveness of these inhibitors in corrosive environments.

### 2.7. Proposed Inhibition Mechanism

Based on experimental and theoretical analyses, an adsorption mechanism can be suggested that describes the interaction between the hydrazone molecules and the iron surface. Experimental and theoretical analyses work in tandem to unveil a comprehensive picture of the adsorption mechanism between hydrazone molecules and the iron surface. Experimental techniques, such as electrochemical measurements and adsorption isotherm analysis, provided macroscopic evidence of inhibitor adsorption and its impact on corrosion behavior. Simultaneously, theoretical approaches like DFTB and molecular dynamics delved into the atomic and molecular level. DFTB calculations revealed the strength and nature of the bonds formed between the inhibitor and iron atoms, pinpointing the inhibitor’s most reactive sites. Molecular dynamics simulations visualized the adsorption process, illustrating the inhibitor molecules’ orientation and dynamic behavior near the iron surface. This powerful combination allowed us to gain a detailed mechanistic understanding of the inhibitor-surface interaction.

It is generally observed that organic molecules containing heteroatoms in their structure readily protonate when immersed in a 15 wt.% HCl solution [55,76]. Concurrently, it has been found that the steel surface acquires a positive charge under similar conditions [77,78]. In this context, due to electrostatic repulsion, molecules can only be adsorbed via pre-adsorbed chloride ions, thereby altering the interfacial charge of the steel to negative. This step, crucial for the success of the corrosion inhibition process by organic molecules, is designated as the physisorption process. As the molecules approach the steel surface, the formation of chemical bonds and charge transfer from the free electron pairs on O and N atoms to the vacant d orbitals of iron are highly probable. This step is considered crucial for the effectiveness of an organic corrosion inhibitor.

**Figure 9:**
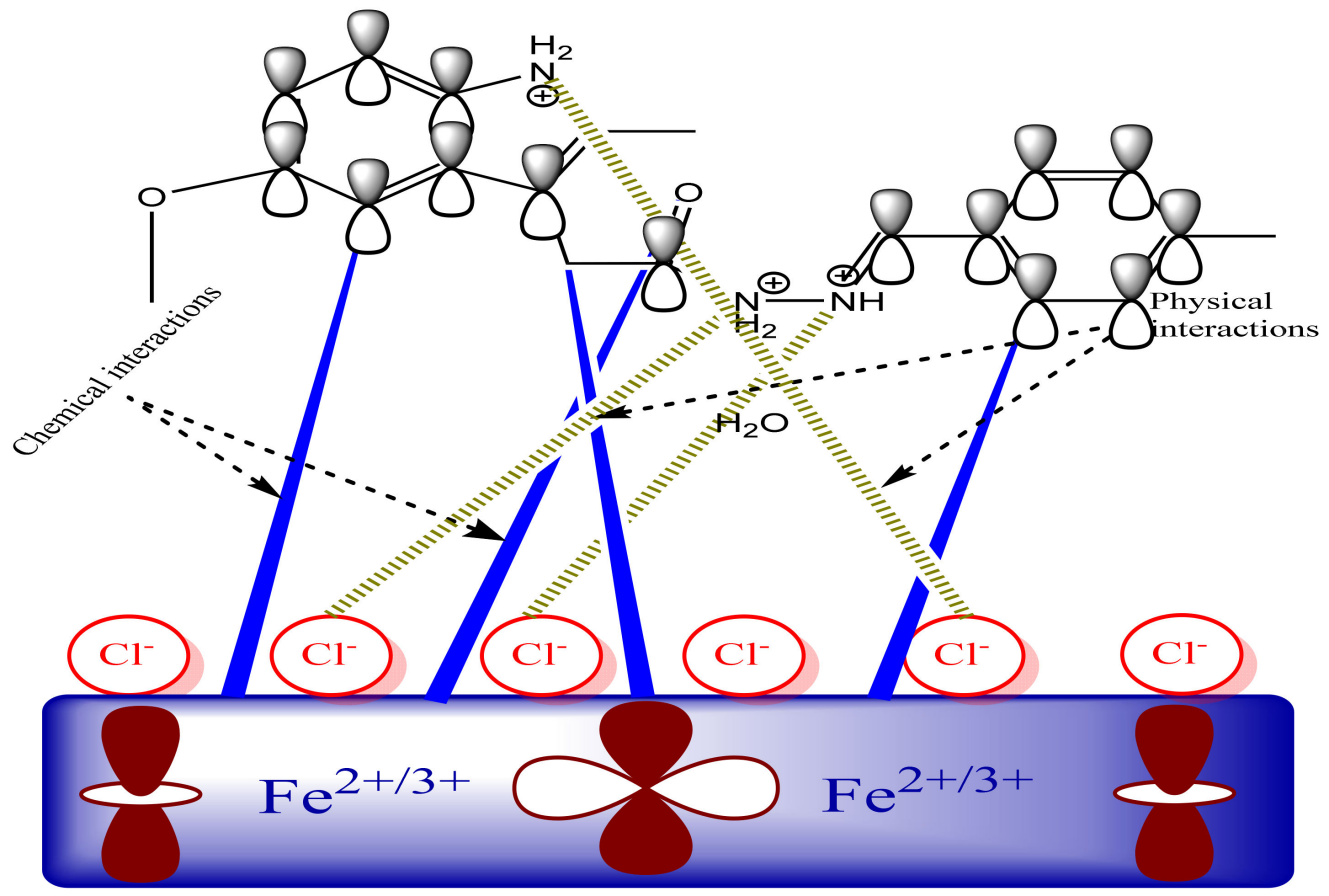
Graphical illustration of the proposed corrosion inhibition mechanism of hydrazones for carbon steel in 15 wt.% HCl. MeHDZ as a reference.

Furthermore, the accumulation of charges on the steel surface can reverse the charge transfer from the steel surface to the antibonding orbitals of the inhibitor molecules, a process known as back-donation [77,78]. These conclusions are consistent with both experimental and theoretical results, which suggest the adsorption of the molecules on the steel surface via both physical and chemical interactions. The proposed mechanism for corrosion inhibition is graphically illustrated in Figure 9.

Overall, this research focuses on understanding how hydrazone derivatives inhibit steel corrosion in aggressive hydrochloric acid (HCl) environments. These inhibitors function by forming a protective barrier on the steel surface, mitigating HCl’s corrosive attack. This protection prevents surface degradation, resulting in improved corrosion resistance. This enhanced durability benefits steel components by extending their lifespan, reducing maintenance costs, and improving the overall safety of structures and equipment operating in acidic environments.

## 3. Materials and Methods

### 3.1. Materials and Electrolyte

In this investigation, the specimens employed were N80 carbon steel, having a composition of 0.31% carbon, 0.19% silicon, 0.92% manganese, 0.01% phosphorus, 0.008% sulfur, and 0.2% chromium, with the balance being iron, all percentages being by mass. The dimensions of these samples were meticulously cut to 1.5 cm in length, 1.2 cm in width, and 0.4 cm in thickness. The experimental setup involved immersing these samples in a corrosive medium, specifically a 15 wt.% HCl solution, obtained by diluting Sigma-Aldrich’s 37% HCl (Burlington, MA, USA) with distilled water. In preparation for the corrosion studies, the steel’s surface was meticulously refined using a series of abrasive papers, progressively moving from a grit of 600 to 1200. The study examined the effects of MeHDZ and HHDZ inhibitors at varying molar concentrations, precisely at 5 × 10^−3^ mol/L, 10^−3^ mol/L, and 5 × 10^−4^ mol/L, ensuring a comprehensive analysis of the corrosion behavior under these conditions.

### 3.2. Synthesis of Hydrazone Compounds

**Scheme 1:**
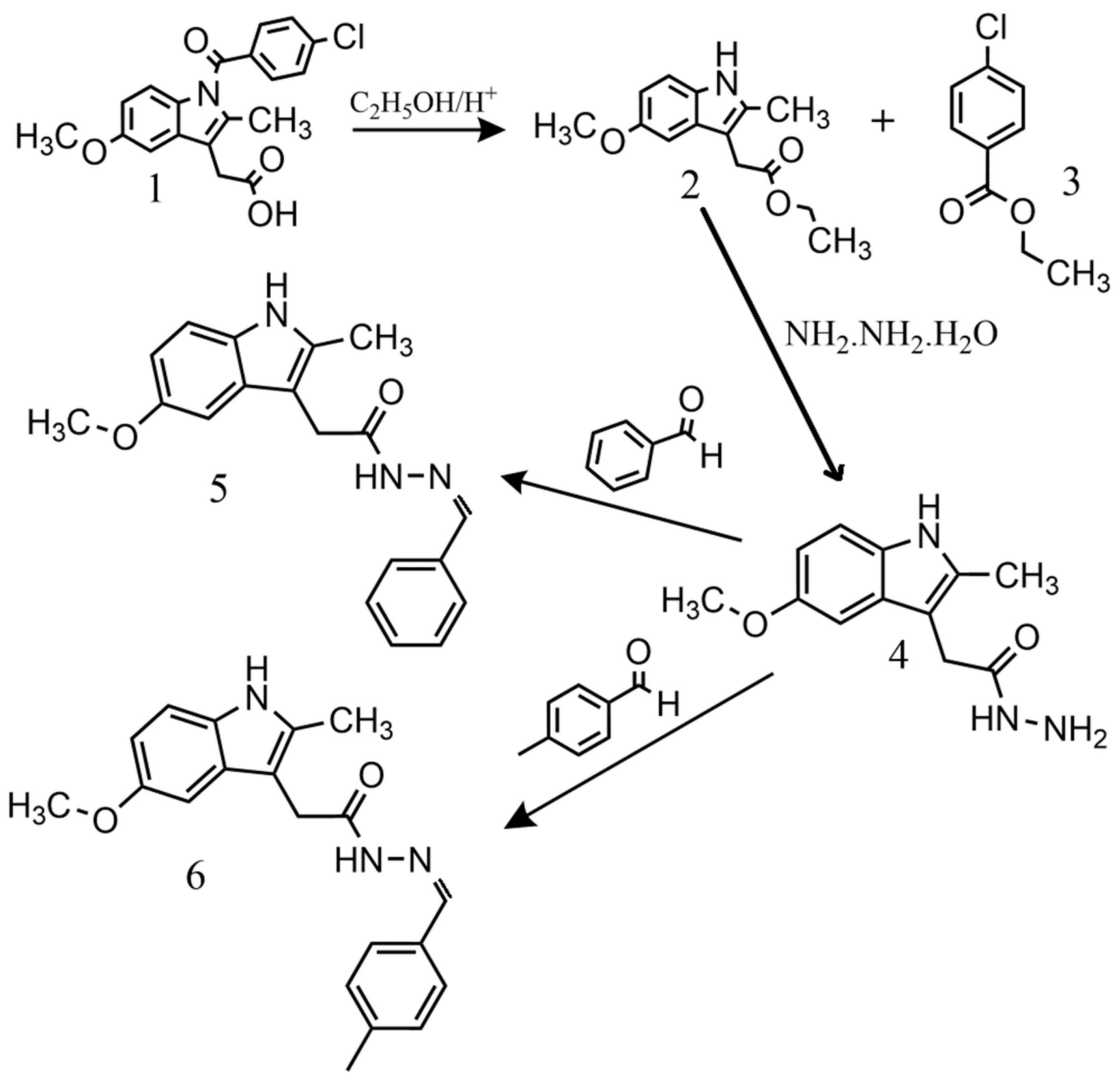
Synthesis procedure for the preparation of hydrazones (**5**; HHDZ) and (**6**; MeHDZ).

This study focuses on the synthesis of two novel hydrazone derivatives, specifically 2-(5-methoxy-2-methyl-1H-indol-3-yl)-N′-[(Z)-phenylmethylidene]acetohydrazide (**5**; HHDZ) and 2-(5-methoxy-2-methyl-1H-indol-3-yl)-N′-[(Z)-(4-methylphenyl)methylidene]acetohydrazide (**6**; MeHDZ), following the outlined scheme and procedures (see Scheme 1 below):

Synthesis of Ethyl (5-Methoxy-2-Methyl-1H-Indol-3-Yl)acetate (**2**): A mixture was prepared by adding absolute ethanol to 0.01 mole of indomethacin and 5 mL of concentrated H_2_SO_4_. This mixture was subjected to reflux for 7 h. Post-reflux, it was poured onto crushed ice and neutralized using NaHCO_3_. The resulting ester, appearing as an oily layer, was extracted via water washing (thrice) and diethyl ether extraction (2 × 50 mL). The compound was finally recrystallized from cyclohexane, yielding silver crystals with a melting point (m.p) of 74–76 °C [80].

IR (KBR): (C=O ester 1710), (NH3316), (C-H aliphatic 2832–2975), (=C-H aromatic 3002) cm^−1^.

Synthesis of 2-(5-Methoxy-2-Methyl-1H-Indol-3-Yl)acetohydrazide (**4**): Compound (**2**) (0.01 mole) was dissolved in ethanol and mixed with 0.06 mole of hydrazine hydrate. The mixture was refluxed for 24 h. Upon cooling, a precipitate formed, which was collected and recrystallized from ethanol, yielding pale yellow crystals (m.p = 166–168 °C [81]). The IR (KBR): (C=O amide 1656), (NH-NH_2_ 3288–3215), (C-H aliphatic 2835–2971) cm^−1^.

General procedure for synthesis of hydrazones (**5**,**6**): In a 100 mL round-bottomed flask (RBF), a reaction mixture was prepared by mixing 0.01 mmole of compound **4** with 0.01 mmole of an appropriate aldehyde and a catalytic amount of glacial acetic acid in 25 mL of absolute ethanol. This mixture was refluxed for 6 h. Post-reflux, the solvent was evaporated, the precipitate collected, and then recrystallized from ethanol, yielding the targeted hydrazones [8].

2-(5-methoxy-2-methyl-1H-indol-3-yl)-N′-[(Z)-phenylmethylidene]acetohydrazide (**5**): m.p = 175–179 °C. IR (KBR): (C=O amide 1641), (-CH=N- 1601), (NH 3182), (C-H aliphatic 2844) cm^−1^. ^1^H-NMR (400 MHz, DMSO): δ = 3.8 (s, 3H, OCH_3_), 2.3 (s, 3H, CH_3_), 3.5 (s, 2H, -CH_2_), 10.6 (s, 1H, NH), 11.2 (s, 1H, NH), 8.2 (s, 1H, -CH=N-), 6.6–7.9 (m, 8H, aromatic protons in two phenyl rings). ^13^C-NMR δ = 13.0 (CH_3_), 31 (-CH_2_), 55 (-OCH_3_), 103–167 (fourteen aromatic carbons in two phenyl rings and pyrrol moiety), 145 (C=N), 173 (C=O amide).

2-(5-methoxy-2-methyl-1H-indol-3-yl)-N’-[(Z)-(4-methylphenyl)methylidene]acetohydrazide(**6**): m.p = 185–189 °C. IR (KBR): (C=O amide 1652), (-CH=N- 1600), (NH 3186), (C-H aliphatic 2898–2835) cm^−1^. ^1^H-NMR (400 MHz, DMSO): δ = 3.8 (s, 3H, OCH_3_), 2.3 (s, 3H, CH_3_), 3.6 (s, 2H, -CH_2_), 10.6 (s, 1H, NH), 11.2 (s, 1H, NH), 8.1 (s, 1H, -CH=N-), 6.7–7.7 (m, 7H, aromatic protons in two phenyl rings). ^13^C-NMR δ = 13.0 (CH_3_), 31 (-CH_2_), 55 (-OCH_3_), 25 (CH_3_), 101–167 (fourteen aromatic carbons in two phenyl rings and pyrrol moiety), 144 (C=N), 173 (C=O amide).

### 3.3. Weight Loss Measurements

In line with the ASTM protocols [82], the investigative framework was meticulously organized, incorporating the precise arrangement and dimensions of the carbon steel samples. These samples underwent corrosion testing within a 15 wt.% HCl solution at controlled temperatures of 303 K and 333 K for 24 h, both in the presence and absence of HHDZ and MeHDZ inhibitors. The examination was conducted in a temperature-stabilized double-walled chamber facilitated by a water thermostat in circulation. Each specimen, positioned in a 250 mL container, was fully immersed in an equal volume of the acidic solution. Following the immersion, the steel samples were rigorously cleansed with distilled water to eliminate any corrosion products and subsequently with acetone, before being dried until a consistent weight was achieved. The variation in weight before and after immersion was recorded as *W* (grams), paving the way for the calculation of the corrosion rate (*C*_WL_) in mm/y utilizing Equation (3) [83].

(3) $$C_{WL}=\frac{K\times W}{A\times \rho \times t}$$

In this formula, *K* symbolizes a constant value (8.76 × 10^4^), ρ signifies the density (7.86 g cm^−3^), A refers to the surface area of the exposed N80 steel in cm^2^, *W* denotes the weight in grams, and t represents the immersion duration in hours.

Subsequently, the parameters of corrosion inhibition efficiency (*η*_wL_) and surface coverage (*θ*) were deduced by analyzing the average corrosion rates, in accordance with Equations (4) and (5):

(4) $$\eta_{wl}=\frac{C_{WL}^{0}-C_{WL}^{inh}}{C_{WL}^{0}}\times 100$$

(5) $$\theta =\frac{\eta_{wl}}{100}$$

Herein, $C_{WL}^{0}$ signifies the corrosion rate in the absence of inhibitors, whereas $C_{WL}^{inh}$ denotes the rate when inhibitors are incorporated.

### 3.4. Electrochemical Measurements

Electrochemical evaluations were executed with the aid of a CS350 model Corrtest Potentiostat/Galvanostat. The apparatus comprised a tri-electrode arrangement: the reference electrode being a saturated calomel electrode (SCE), the counter electrode composed of platinum wire, and the N80 steel electrode (1 cm^2^) serving as the working electrode, positioned in close proximity to the reference electrode. The working electrode underwent immersion in 80 mL of the corrosive solution, followed by a stabilization period of 30 min at a temperature of 303 ± 2 K to achieve equilibrium at the open circuit potential (OCP). Electrochemical impedance spectroscopy (EIS) was performed by imposing a disturbance of 10 mV amplitude at the equilibrated OCP over a frequency range spanning from 100 kHz to 10 mHz. The EIS data procured were analyzed using a corresponding electrical circuit model, formulated within Z-View software Ver. 2. The inhibition efficiency based on EIS (*η*_EIS_) was ascertained employing Equation (6):

(6) $$\eta_{EIS}(\%)=\frac{R_{p}^{inh}-R_{p}^{0}}{R_{p}^{inh}}\times 100$$

where $R_{p}^{inh}$ and $R_{p}^{0}$ symbolize the polarization resistance metrics in the presence and absence of the inhibitor, respectively.

In the context of potentiodynamic polarization assessments, the methodology involved scanning the potential from −650 to −150 mV against SCE at a scan rate of 0.5 mV/s. This specific range of potential was selected to conduct a comprehensive investigation into both the anodic and cathodic reactions of N80 steel in 15 wt.% HCl. The inhibition efficacy in potentiodynamic polarization (*η*_PDP_) was calculated according to Equation (7):

(7) $$\eta_{PDP}\left(\%\right)=\frac{i_{corr}^{0}-i_{corr}^{inh}}{i_{corr}^{0}}\times 100$$

wherein $i_{corr}^{0}$ signifies the corrosion current density without inhibitors, and $i_{corr}^{inh}$ denotes the density when inhibitors are added.

### 3.5. SEM Analysis

The surface morphology of N80 carbon steel samples, following a 24-h immersion at 303 K in solutions containing the most efficient inhibitor (MeHDZ) as well as in inhibitor-free solutions, was meticulously examined using Scanning Electron Microscopy (SEM) (JEOL Ltd., Tokyo, Japan). Post-immersion, the samples were extensively cleansed using double-distilled water and subsequently dried. For the surface structure analysis, SEM imaging was conducted using the MIRA3 model by TESCAN, based in the Brno, Czech Republic, operating at a voltage of 15 kV.

### 3.6. SCC-DFTB Simulations

In this study, the Self-Consistent Charge Density Functional Tight-Binding (SCC-DFTB) method was employed, leveraging its capability to simulate electronic and structural attributes with a precision akin to first-principle Density Functional Theory (DFT) simulations, yet significantly faster, especially for sizable systems. Specifically, the spin-polarized version of SCC-DFTB was utilized, integrating dispersion interactions (with the assistance of Slater–Koster trans3d parameters) via the DFTB+ software, interfaced through Materials Studio Ver. 7 [84]. This approach was used to meticulously analyze the interactions between hydrazone molecules and iron surfaces.

The optimization of these adsorption systems was methodically carried out, enforcing a stringent SCC convergence criterion of 10^−8^ atomic units. Thermal smearing was introduced through the Methfessel–Paxton distribution, while the Broyden mixing algorithm facilitated the convergence process. The remaining convergence parameters adhered to the high-quality standards set by DFTB^+^. Simultaneously, the bulk lattice dimensions were meticulously refined employing an (8 × 8 × 8) k-point grid, culminating in an optimized lattice parameter of 2.877 Å. This measurement closely aligns with the experimental lattice parameter of 2.862 Å, validating the precision of the chosen computational parameters.

For the optimization of Hydrazone-Fe(110) interface systems, convergence was achieved with a (2 × 2 × 1) k-point grid. The adsorption models were created by constructing an Fe(110) surface using a (5 × 5) supercell and introducing a 20 Å vacuum buffer along the z-axis to mitigate interactions with periodic images in all dimensions. The hydrazone molecules were carefully positioned on the topmost layer of the slab, with all atoms, barring the two lowermost atomic layers, granted freedom to relax. The simulations proceeded with the hydrazone molecules covering 1/25 of the monolayer (ML) on the surface. This study focused on the parallel orientation of hydrazones on the Fe(110) surface, positing it as the optimal balance between energy stability and effective surface coverage, although other adsorption configurations are possible. The standalone molecular structures were optimized using SCC-DFTB within a cubic domain of 40 Å. The interaction energy between the hydrazones and the Fe(110) surface was quantified using the equation:

(8) $$E_{inter}=E_{mol/surf}\;-\;\left(E_{mol}+\;E_{surf}\right)$$

Here, $E_{mol}$, $E_{surf}$, and $E_{mol/surf}$ represent the total energies of the isolated molecules, the Fe(110) iron surface, and the combined molecule/Fe(110) adsorption systems, respectively.

### 3.7. Molecular Dynamics (MD) Simulations

Molecular dynamics (MD) simulations serve as an essential tool for examining the adsorption patterns and interactions of corrosion inhibitors on steel surfaces. In this research, the Materials Studio software Ver. 7, featuring the Forcite module, was utilized for the simulations. A supercell (10 × 10), extracted from the pure iron along the (110) plane, formed a simulation box with a bilayer structure. The amorphous cell module was applied to introduce solvent layers containing an inhibitor (either HHDZ or MeHDZ), along with 500 water molecules, 9 hydronium (H_3_O^+^) ions, and 9 chloride (Cl^−^) ions. The simulation setup, after an initial phase of structural optimization, was subjected to MD simulations spanning 5000 ps. These simulations were conducted with a timestep of 1 fs within the NVT ensemble, using the COMPASSIII force field. For all other parameters within the Forcite module, the ‘fine’ setting was selected.

## 4. Conclusions

This study successfully integrated experimental techniques (weight loss, potentiodynamic polarization, and electrochemical impedance spectroscopy) with advanced computational methods (SCC-DFTB and molecular dynamics simulations) to evaluate the corrosion inhibition capabilities of two novel hydrazone derivatives on N80 steel in 15 wt.% HCl. Key findings include:

- MeHDZ and HHDZ achieved remarkable inhibition efficiencies of 98% and 94%, respectively, at 5 × 10^−3^ mol/L (EIS analysis).
- MeHDZ demonstrated impressive stability over a 30-h immersion period. Its polarization resistance increased from 547 Ohm cm^2^ (initial) to 560 Ohm cm^2^, highlighting its ability to provide long-term corrosion mitigation.
- The inhibitors’ behavior aligned with the Langmuir adsorption isotherm, suggesting the formation of a uniform, protective layer on the steel surface.
- SEM analysis revealed smoother surfaces in the presence of inhibitors, visually confirming their adsorption and protective action.
- Computational simulations corroborated experimental findings, indicating the formation of chemical bonds between inhibitor molecules and iron atoms on the steel surface. A predominantly parallel adsorption mode was computationally predicted.

## Figures and Tables

**Table 1:** Corrosion rates and inhibition efficiency for carbon steel in 15 wt.% HCl with varying concentrations of MeHDZ and HHDZ over 24 h at 303 K and 333 K.

[Inhibitor] (mol/L)	W (mm/year)		*η* (%)	
	303	333	303	333
15 wt.% HCl	23.7	253.4	-	-
MeHDZ				
5 × 10^−3^	0.850	23.920	96.41	90.56
1 × 10^−3^	1.744	32.080	92.64	87.34
1 × 10^−4^	2.315	39.505	90.23	84.41
HHDZ				
5 × 10^−3^	1.509	31.218	93.63	87.68
1 × 10^−3^	2.355	39.631	90.06	84.36
1 × 10^−4^	2.744	45.966	88.42	81.86

**Table 2:** Adsorption characteristics for the corrosion of carbon steel in the presence of two compounds, MeHDZ and HHDZ, at a temperature of 303 K.

Inhibitor	Slope	$K_{ads}$ (L/mol)	${∆G}_{ads}^{{}^{\circ}}$ (KJ/mol)
MeHDZ	1.05	39,985	−36.79
HHDZ	1.02	40,706	−36.83

**Table 3:** Electrochemical parameters from PPC for carbon steel in 15 wt.% HCl without and with different concentrations of MeHDZ and HHDZ at 303 K.

[Inhibitor] (mol/L)	*i*_corr_ (µA/cm^2^)	−*E*_corr_ (mV vs SCE)	*β*_a_ (mV/dec)	−*β*_c_ (mV/dec)	*η* (%)
15% HCl	1711	441	90	111	-
MeHDZ					
1 × 10^−4^	245	407	81	151	89
1 × 10^−3^	136	404	75	143	92
5 × 10^−3^	95	416	79	134	94
HHDZ					
1 × 10^−4^	243	408	77	150	86
1 × 10^−3^	168	416	74	147	90
5 × 10^−3^	118	400	78	139	93

**Table 4:** EIS parameters for carbon steel in 15 wt.% HCl without and with different concentrations of MeHDZ and HHDZ at 303 K.

[Inhibitor] (mol/L)	*R*_s_ (Ω cm^2^)	*R*_p_ (Ω cm^2^)	CPE		*C*_eff,dl_ (µF/cm^2^)	*η* (%)
			*Q*_dl_ (µF/cm^2^)	n		
15% HCl	1.5	5.51	697.8	0.837	236.2	-
MeHDZ						
1 × 10^−4^	1.9	88	159	0.795	52.8	93
1 × 10^−3^	2.1	157	96	0.831	40.9	96
5 × 10^−3^	1.7	547	69	0.827	34.7	98
HHDZ						
1 × 10^−4^	1.7	46	216	0.814	75.3	88
1 × 10^−3^	1.5	55	165	0.836	65.6	89
5 × 10^−3^	1.8	101	122	0.808	43.0	94

**Table 5:** Values of electrochemical parameters determined from the EIS plots for carbon steel immersed in a 15 wt.% HCl Solution with 5 × 10^−3^ mol/L of MeHDZ at 303 K, taken at various immersion durations.

Time (h)	*R*_s_ (Ω cm^2^)	*R*_p_ $(Ω\;{cm}^{2}$)	n	*Q* (µF/cm^2^)	*C*_eff,dl_ (µF/cm^2^)
0.5	1.7	547	0.827	69	34.7
3	1.1	479	0.836	71	43
6	2.1	515	0.817	68	44
12	2.4	544	0.813	73	49
24	1.6	576	0.849	72	49
30	2.1	560	0.801	67	41

## Data Availability

The data cannot be shared at this time as the data also forms part of an ongoing study.
